# Burden and Determinant of Inadequate Dietary Diversity among Pregnant Women in Ethiopia: A Systematic Review and Meta-Analysis

**DOI:** 10.1155/2020/1272393

**Published:** 2020-08-14

**Authors:** Hagos Degefa Hidru, Meresa Berwo Mengesha, Yared Hailesilassie, Fissaha Tekulu Welay

**Affiliations:** ^1^College of Medicine and Health Sciences, Department of Public Health, Adigrat University, Adigrat, Ethiopia; ^2^College of Medicine and Health Sciences, Department of Midwifery, Adigrat University, Adigrat, Ethiopia; ^3^College of Medicine and Health Sciences, Department of Nursing, Adigrat University, Adigrat, Ethiopia

## Abstract

**Background:**

Inadequate dietary diversity intake during pregnancy results in increased risks of negative maternal and perinatal outcomes. About one million neonates die on the first day of life due to inadequate dietary intake during pregnancy as a result of maternal complication and adverse birth outcomes. This review summarizes the burden of inadequate dietary diversity and its determinants among pregnant women at the national level of Ethiopia.

**Methods:**

Studies were retrieved from selected electronic databases, including PubMed, Cochrane Library, and Google Scholar. Random-effects model meta-analysis was used to estimate the pooled burden of inadequate dietary diversity and its determinants at a 95% confidence interval with its respective odds ratio (OR) using statistical R-software version 3.6.1. Moreover, quality appraisal of the included studies, publication bias, and level of heterogeneity were checked with subgroup analysis and sensitivity influential test. The searches were restricted to articles published in the English language only, and Medical Subject Headings (MeSH terms) was used to help expand the search in advanced PubMed search.

**Result:**

A total of 850 articles were identified through the initial search of which 21 studies were included in the final review yielding a total sample size of 9,230 pregnant women. The pooled prevalence of inadequate dietary diversity was 53% (95% CI: 44%, 62%). Food insecurity [AOR = 2.18, (95% CI: 1.02, 4.63)], family size of greater than four [AOR = 1.46, (95% CI: 1.10, 1.95)], rural residence [AOR = 4.52, (95% CI: 1.02, 20.09)], no formal educational status [AOR = 4.50, (95% CI: 1.02, 20.09)], and a lack of counseling about dietary diversity [AOR = 2.75, (95% CI: 2.17, 3.48)] were among the significantly associated factors for inadequate dietary diversity.

**Conclusion:**

In this review, there was a high prevalence of inadequate dietary diversity among pregnant women at the national level in Ethiopia. Therefore, strengthening early counseling and diagnosis of dietary intake and undernutrition during the antenatal care period is important.

## 1. Introduction

Dietary diversity refers to an increase in the variety of foods across and within food groups [1, 2]. Inadequate monotonous diets for women of reproductive age, who are often nutritionally vulnerable because of the physiological demands of pregnancy, are very common. Insufficient nutrient intakes before and during pregnancy can affect both women and their infants [3, 4].

Dietary diversity in pregnant women is not only critical to reducing maternal mortality but morbidity and is a foundation to the developing fetus' growth and reduced perinatal outcome complications [5, 6]. Inadequate dietary diversity intake during pregnancy results in increased risks such as intrauterine growth restriction (IUGR), abortion, low birth weight, preterm birth, and prenatal and infant mortality and morbidity [7–9].

Many women in sub-Saharan Africa including Ethiopia remain particularly exposed to inadequate intake of micronutrients, resulting in different types of malnutrition and its complications. Seven percent of the global disease burden and at least one-fifth of maternal deaths and poor maternal outcomes are as the result of maternal inadequate dietary diversity [10, 11]. About one million neonates die on the first day and in the first week of life due to inadequate dietary intake of the mothers prior to and during pregnancy, as linked with increasing trends in maternal anemia, mortality, and adverse birth outcomes [12].

Although insufficient attention has been given to the extent, causes, and consequences of such nutritional deficiency, the lack of dietary diversity in pregnant women in Ethiopia remains a leading cause of maternal and perinatal complications. Ethiopia has a wide range of agroclimatic conditions and grows a variety of cereals, root crops, and vegetables; however, money of these are not fully utilized because of maternal and environmental factors, dependency on single food crops, inadequate antenatal care (ANC) follow-up, unwanted pregnancy, maternal knowledge, lower socioeconomic health status, and season variability [12].

The prevalence of inadequate dietary diversity and its determinant variables among pregnant women is inconsistent across regions resulting in variabilities and uncertainties over time among different studies done at the national level of Ethiopia. In addition to this gap, lack of documented and published peer-reviewed data on the pooled prevalence of dietary diversity and its determinants among pregnant women at the national level in Ethiopia hinders program managers in designing and implementing effective strategies. With there being minimal existing evidence in developing countries, including Ethiopia, evidence based on pooled results at the national level on burden and determinant of inadequate dietary diversity is needed to guide decision-making and implementation programs. Therefore, this review would pool the evidence of inadequate dietary diversity and its determinants among pregnant women at the national level of Ethiopia.

## 2. Methods

### 2.1. Searching Strategies

We systematically reviewed and analyzed published research articles to determine the pooled prevalence of inadequate dietary diversity and its determinants among pregnant women in Ethiopia. We identified and searched published articles using major electronic databases, including PubMed, Cochrane Library, JURN, Hinari library, AJOL (African Journal Online), Google Scholar, and Science Open, as well as a manual search from grey literature was conducted accordingly. The key terms/phrases employed used for PubMed search were “Prevalence” OR “Incidence” OR “Burden” AND “Determinant” OR “Predictors” OR “Factors” AND “Insecurity” OR “Undernutrition” OR “Diet” OR “Diversity” OR “Inadequate” AND “Pregnant” OR “Reproductive age” AND “Women” AND “Ethiopia'‘. The searches were restricted to articles published in the English language only. The search terms were predefined to allow a comprehensive search strategy that included all fields within records, and Medical Subject Headings (MeSH terms) was used to help expand the search in advanced PubMed search. This study also used Boolean operators (we combined keywords with the “OR” operator and then linked the search strategies with the “AND” operator).

### 2.2. Eligibility Criteria

We reviewed articles from the initial search using defined inclusion and exclusion criteria.

### 2.3. Inclusion Criteria

All studies which focused on the burden of inadequate dietary diversity and its determinants among pregnant women across Ethiopia were included in this systematic review and meta-analysis. Both community- and facility-based cross-sectional and cohort studies irrespective of their size were included. Articles published from 2013 until 2019 in the English language were included.

### 2.4. Exclusion Criteria

Studies that did not report specific outcomes either for the burden of inadequate dietary diversity, determinants of dietary diversity, or both quantitatively were excluded from this systematic review and meta-analysis. These papers which could not be fully accessed at the time of our search process were excluded from this review after a contact was attempted with the principal investigator through e-mail on two occasions.

### 2.5. Data Extraction and Synthesis

The database search results were combined, and duplicate articles were removed using EndNote (version X8). Data were extracted by two authors using a standardized data extraction spreadsheet. Data extraction spreadsheet was pretested on five randomly selected articles, and modification was done accordingly. The spreadsheet included study characteristics such as authors' name, regions, publication year, total sample size, sampling technique, study design, prevalence of dietary diversity (adequate and inadequate), and data also extracted for determinants of respective studies with their frequency of adequate and inadequate dietary diversity including family size, residence, age group, monthly income, women education, food insecurity, and counseling about dietary diversity during ANC follow-up were extracted from each article.

The initial screening of the articles by title, abstract, and full text was carried out by two authors (HDH and MM) independently based on the predefined inclusion and exclusion criteria. After each screening round (title, abstract, and full texts), the authors met and resolved any discrepancies by discussion, while potential disagreements were solved by the involvement of the third authors (YH and FT). The reference lists of the included full-text articles were appraised to ascertain additional articles of relevance to determine necessity of retrieving the full text. Finally, all the included accessible full-text articles were extracted to assemble appropriate information based on the inclusion criteria.

### 2.6. Quality Assessment

The quality of all the included articles was assessed for their risk bias using Joanna Briggs Institute Meta-Analysis of Statistics Assessment and Review Instrument (JBI-MAStARI) [13] adapted for cross-sectional and cohort study design. Two independent reviewers critically appraised each paper. Studies which scored between five and nine were included in the final systematic review and meta-analysis. The items included (1) eligibility criteria, (2) representativeness to the target population, (3) sampling technique, (4) measurable objective, (5) data collection directly from subjects, (6) controlling confounding, (7) reliability and validity of exposure and outcome variable, (8) appropriate data analysis, (9) length of prevalence period, and (10) appropriateness of numerator and denominator. Each item was assessed as either yes (scored 1), no (scored 0), and then weighted the total score out of nine and ten for cross-sectional and cohort study, respectively (Table 1).

### 2.7. Outcome Measurement

The primary aim was to know the pooled prevalence of inadequate dietary diversity among pregnant women at the national level, while the secondary aim of this review was to identify different factors affecting inadequate dietary diversity among pregnant women in Ethiopia. By aggregating these two levels of information, it will build understanding for future implemented for a policy intervention program in order to decrease the high burden of maternal mortality and perinatal outcome complication.

### 2.8. Strategy of Data Synthesis and Analysis

Burden of inadequate dietary diversity and estimates for risk factors obtained from each study were determined as a single estimate pooled. The extracted data were entered into the computer via an Excel sheet for screening their title, abstract, and full texts and then exported to R-statistical software version 3.6.1 for analysis. Evidence of publication bias was assessed using visual inspection of funnel symmetry test, sensitivity test, and Eggers regression test, and trim fill analysis for the asymmetric funnel was conducted. The heterogeneity test across studies was done using the inverse variance (*I*^2^) with the Cochrane *Q* statistic test. Forest plots to visualize heterogeneity for the pooled prevalence using the random-effect model and *p* value less than 0.05 to determine heterogeneity were also conducted. Risk factors obtained from each primary study were thematically organized, and their effect sizes were pooled accordingly using the random-effect model. In case of significant heterogeneity using visual forest plots in the random-effect model along with 95% confidence interval, we did subgroup analysis and sensitivity test of influential test and we did not get significant variation on the value of heterogeneity. Subgroup analysis was conducted using sampling technique, study design (facility and community-based cross-sectional), publication year, sample size, and regions and no changed value on the heterogeneity was observed except for the sample size variation. The Preferred Reporting Items for Systematic Reviews and Meta-Analysis (PRISMA) statement for reporting a systematic review and meta-analysis was used to clearly present the study inclusion, exclusion, and rationales.

## 3. Result

**Figure 1:**
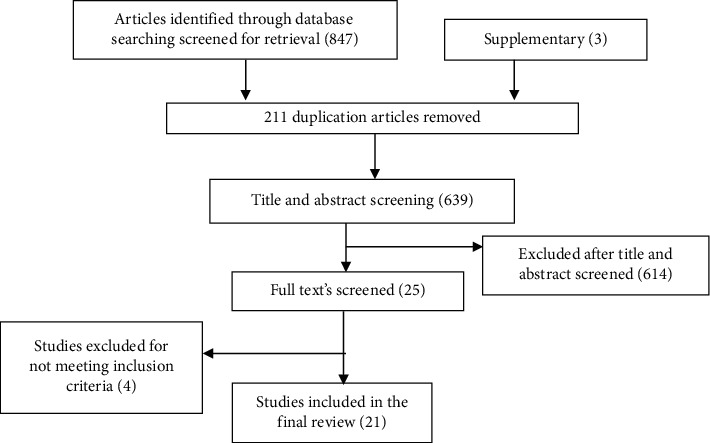
Flowchart shows selected and screening of articles for systematic review and meta-analysis.

In this review, we extracted data related to dietary diversity and their determinants among pregnant women at the national level. A total of 850 articles were retrieved from different sources through the electronic database (847) and supplementary from unpublished (3) searches of which 211 duplicated articles were excluded. From the remaining 639 articles, 614 articles were excluded after reading their titles and abstracts based on the predefined inclusion criteria. Finally, 25 full-text articles were accessed and assessed for eligibility criteria. Based on the predefined criteria and after critical appraisal, only 21 articles were included for the final systematic review and meta-analysis (Figure 1).

### 3.1. Characteristics of Included Studies

A total of twenty-one articles had met the inclusion criteria. All the included studies were published between 2013 and 2019 in the English language. Twenty facility, community-based cross-sectional and one cohort studies were included using an estimated sample size ranging from 153 [25] up to 759 [29] pregnant women between 2015 and 2019, respectively. A total sample of 9,230 pregnant women was included to estimate the pooled prevalence of dietary diversity and its associated factors among pregnant women at the national level (Table 1). Of the total 21 articles, about half were conducted in Amhara regional state, seven studies in Oromia regional state, two studies in Southern Nation Nationality and People regional state, one study each in at Tigray regional state, Gambella regional state, and in Dire Dawa administrative state (Table 1).

### 3.2. Prevalence of Inadequate Dietary Diversity in Ethiopia (Meta-Analysis)

**Figure 2:**
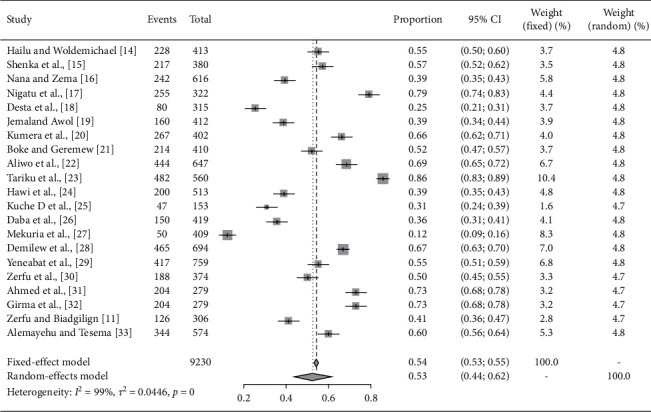
Forest plot showing the pooled prevalence of inadequate dietary diversity among pregnant women in Ethiopia from 2013 up to 2019.

The pooled prevalence of inadequate dietary diversity among pregnant women at the national level in Ethiopia was found to be 53% (95% CI: 44%, 62%), using visual forest plots in the random-effect model along with 95% confidence interval (Figure 2). Level of heterogeneity was checked by using subgroup analysis and sensitivity influential test, and we did not get significant variation in the value of heterogeneity. Publication bias was assessed using the visual funnel symmetric test, Egger test, and trim fills analysis in addition to the critical appraises of individuals risk bias.

### 3.3. Associated Factors of Inadequate Dietary Diversity among Pregnant Women in Ethiopia

**Figure 3:**
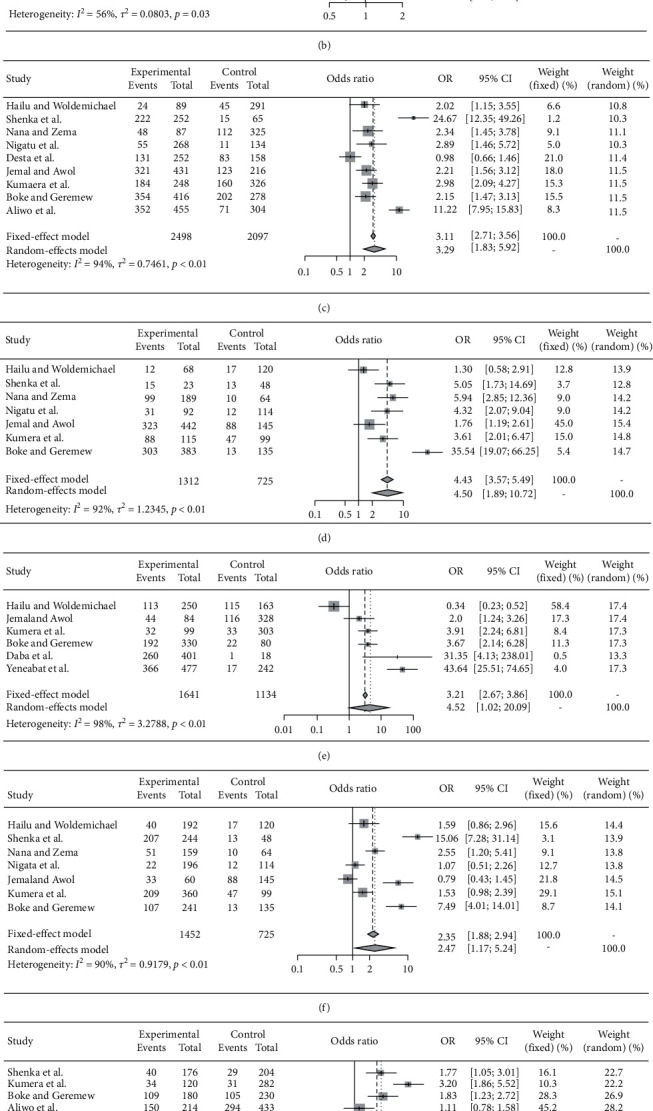
Forest plot which describe the pooled association factors of inadequate dietary diversity among pregnant women in Ethiopia from 2013 up to 2019: (a) inadequate dietary diversity and food insecurity; (b) inadequate dietary diversity and family size; (c) inadequate dietary diversity and income status; (d) inadequate dietary diversity and with no formal education; (e) inadequate dietary diversity and rural residence; (f) inadequate dietary diversity and primary and secondary educational levels; (g) inadequate dietary diversity and age group; (h) inadequate dietary diversity and counseling.

Family size, educational status, food insecurity, economic status, residence, history of counseling about dietary diversity, and age group were the variable found to have a significant association with the occurrence of inadequate dietary diversity at the national level. The odds of inadequate dietary diversity were 2.18 times higher among food-insecure pregnant women compared with food-secure pregnant women [AOR = 2.18, (95% CI: 1.02, 4.63)] (Figure 3(a)). The odds of inadequate dietary diversity were 1.46 times higher with family size greater than four compared with pregnant women whose family size was less than four [AOR = 1.46, (95% CI: 1.10, 1.95)] (Figure 3(b)). The odds of inadequate dietary diversity were 3.29 times higher among pregnant women with their monthly income of less than 1500 Ethiopian Birr compared to those pregnant women with their monthly income ≥1500 Ethiopian Birr [AOR = 3.29, (95% CI: 1.83, 5.92)] (Figure 3(c)). The odds of inadequate dietary diversity were 4.50 times higher among pregnant women with no formal education compared to those pregnant women with their educational status college and above [AOR = 4.50, (95% CI: 1.89, 10.72)] (Figure 3(d)). The odds of inadequate dietary diversity were 4.52 times higher among rural residence pregnant women compared with pregnant women who reside in urban [AOR = 4.52, (95% CI: 1.02, 20.09)] (Figure 3(e)). The odds of inadequate dietary diversity were 2.47 times higher among pregnant women with primary and secondary educational status compared to those pregnant women with educational status as college level and above [AOR = 2.47, (95% CI, 1.17, 5.24)] (Figure 3(f)). The odds of inadequate dietary diversity were 1.79 times higher among pregnant women less than 25 years of age compared with their older counterparts [AOR = 1.79, (95% CI: 1.17, 2.73)] (Figure 3(g)). The odds of inadequate dietary diversity were 2.75 times higher among those pregnant women who did not get counseling about dietary diversity during their antenatal care compared with pregnant women who get their dietary counseling during their antenatal care [AOR = 2.75, (95% CI: 2.17, 3.48)] (Figure 3(h)).

## 4. Discussion

The finding of this review revealed that more than half of pregnant women were found with inadequate dietary diversity in Ethiopia. During the meta-analysis, potential sources of heterogeneity were investigated using the sensitivity influential test, and subgroup analysis on the sampling size, regions, sampling technique, facility- and community-based cross-sectional study, and year of publication was conducted.

The source of heterogeneity in this review might be due to the difference in sample size with in the individual articles because as the two extreme sample size (the lowest and largest size) was removed based on the sensitivity analysis, the value of heterogeneity was changed. The pooled prevalence of inadequate dietary diversity among pregnant women at the national level in the current systematic review and meta-analysis was found to be 53% from the random-effect model, which is higher than prevalence reported in studies conducted in Kenya (37%, 39.4%) [34, 35], France (36%) [36], studies conducted in four low-middle income countries (Guatemala, India, Pakistan, and Democratic Republic of the Congo (30%)) [10], Ghana (14.5%) [37], Indonesia (40%) [38], Pakistan low dietary diversity (5%) (39), Nigeria (16.5%) [39], and Ghana (48.8%) [40]. Our findings are lower than reports from Kenya (94.5%) [41] and Algeria (68%) [42]. These variations might be due to difference in sample size, measurement items of dietary diversity and the cut point to say inadequate dietary diversity from country to country, difference in study period (seasonal difference), difference in the commonly utilized available diet in their place of residence, geographical location variation, and cultural and/or religious aspects of being pregnant as well knowledge differences in ways of access to information on dietary diversity, educational level, economical status, family size, number of parity, residence, food security status, difference on counseling, and might be due to age of pregnancy. The pooled finding of this review was similar to studies performed in Ghana (52%) [43] and in Nigeria (50%) [44], which may relate to similarity in the diet measurement item that was used to assess inadequate dietary diversity during pregnancy, similar availability of diet, might similar access of information, and knowledge of pregnancy-related diet.

### 4.1. Strengths and Limitations

The strength of the current review lies in the international standardized guidelines on the conduct and reporting of systematic review and meta-analysis at the national level of Ethiopia. Though searching was done for unpublished papers and analytical studies (cohort and case-control studies), only published studies and most of the cross-sectional study design were included because we did not find any published case-control studies through the searching engine we had used.

## 5. Conclusion and Recommendation

The pooled result of this systematic review and meta-analysis showed a high prevalence of inadequate dietary diversity among pregnant women at the Ethiopia national level in which more than half of pregnant women were found to lack sufficient dietary diversity. The review had found that factors like food insecurity, lack of counseling, low economic status, no formal, primary, and secondary educational status, family size, and rural residence were positively significant associated with the occurrence of inadequate dietary diversity among pregnant women in Ethiopia. The value of high heterogeneity might be due to the sample size variation among the individual studies.

Therefore, the responsible stakeholders and governmental health officers should strengthen the system and procedures for early counseling about the importance of dietary diversity for pregnancy and perinatal outcomes. To alleviate the inadequate dietary problem among pregnant women at the national level in Ethiopia, the government should coordinate and integrate dietary diversity programs at all levels with different sectors working on the maternal and nutritional services. There should also be strict and frequent monitoring of their nutritional status during antenatal follow-up than previous and should work in collaboration with agricultural sectors especially for the rural settings in order to avail themselves on dietary access in terms of quality and quantity to minimize inadequate dietary diversity burdens and to strengthen the availability and utilization of the service that is available on each health facilities.

## Figures and Tables

**Table 1:** Characteristics of included studies to estimate pooled prevalence of inadequate dietary diversity and its determinants among pregnant women in Ethiopia from 2013 up to 2019.

Id no.	Author	Publication year	Region	Study design	Sampling technique	Sample size	Frequency dietary diversity		Quality score
							Inadequate	Adequate	
1	Hailu and Woldemichael [14]	2019	Oromia	FCS	Systematic sampling	413	228	185	6
2	Shenka et al. [15]	2018	Dire Dawa	FCS	Systematic sampling	380	217	163	5
3	Nana and Zema [16]	2018	Amhara	CCS	Cluster sampling	616	374	242	8
4	Nigatu et al. [17]	2018	Gambela	CCS	Simple random sampling	322	255	67	5
5	Desta et al. [18]	2019	Oromia	FCS	Systematic sampling	315	80	235	5
6	Jemal and Awol [19]	2019	Tigray	FCS	Systematic sampling	412	160	252	6
7	Kumera et al. [20]	2018	Amhara	FCS	Systematic sampling	402	267	135	6
8	Boke and Geremew [21]	2018	SNNP	CCS	Simple random sampling	410	214	196	6
9	Aliwo et al. [22]	2019	Amhara	CCS	Cluster sampling	647	444	203	9
10	Tariku et al. [23]	2019	Amhara	Cohort	_	560	482	78	7
11	Hawi et al. [24]	2018	Oromia	FCS	_	513	200	313	6
12	Kuche et al. [25]	2015	SNNP	CCS	Cluster sampling	153	47	106	5
13	Daba et al. [26]	2013	Oromia	FCS	Systematic sampling	419	150	269	6
14	Mekuria et al. [27]	2017	Amhara	CCS	Systematic sampling	403	50	353	6
15	Demilew et al. [28]	2019	Amhara	CCS	Cluster sampling	694	465	229	9
16	Yeneabat et al. [29]	2019	Amhara	CCS	Multistage sampling	759	417	342	9
17	Zerfu et al. [30]	2019	Oromia	FCS	Simple random sampling	374	188	186	5
18	Ahmed et al. [31]	2018	Amhara	FCC	_	279	204	75	5
19	Girma et al. [32]	2019	Oromia	FCC	Consecutive sampling	279	204	75	5
20	Zerfu and Biadiglign [11]	2018	Oromia	FCC	Systematic sampling	306	126	180	6
21	Alemayehu and Tesema [33]	2016	Amhara	CCS	Cluster sampling	574	344	230	8

CCS = community-based cross-sectional; FCS = facility-based cross-sectional; SNNP = South Nations Nationalities and Peoples.

## Abbreviation

ANC: Antenatal care
AOR: Adjust odds ratio
CI: Confidence interval
FAO: Food and Agricultural Organization
IUGR: Intrauterine growth restriction
JBI: Joanna Briggs Institute
MeSH: Medical Subject Headings
NACS: Nutritional Assessment Counseling and Support
PRISMA: Preferred Reporting Items for Systematic Review and Meta-Analysis
SNNP: South Nations Nationalities and Peoples
WHO: World Health Organization.
